# Functional Networks of Nucleocytoplasmic Transport-Related Genes Differentiate Ischemic and Dilated Cardiomyopathies. A New Therapeutic Opportunity

**DOI:** 10.1371/journal.pone.0104709

**Published:** 2014-08-19

**Authors:** María Micaela Molina-Navarro, Juan Carlos Triviño, Luis Martínez-Dolz, Francisca Lago, Jose Ramón González-Juanatey, Manuel Portolés, Miguel Rivera

**Affiliations:** 1 Cardiocirculatory Unit, Health Research Institute Hospital La Fe, Valencia, Spain; 2 Sistemas Genómicos, Valencia, Spain; 3 Heart Failure and Transplantation Unit, Cardiology Department, La Fe University Hospital, Valencia, Spain; 4 Cellular and Molecular Cardiology Research Unit, Department of Cardiology and Institute of Biomedical Research, University Clinical Hospital, Santiago de Compostela, Spain; Indiana University, United States of America

## Abstract

Heart failure provokes alterations in the expression of nucleocytoplasmic transport-related genes. To elucidate the nucleocytoplasmic transport-linked functional network underlying the two major causes of heart failure, ischemic cardiomyopathy (ICM) and dilated cardiomyopathy (DCM), we examined global transcriptome profiles of left ventricular myocardium tissue samples from 31 patients (ICM, n = 10; DCM, n = 13) undergoing heart transplantation and control donors (CNT, n = 8) using RNA-Sequencing and GeneMANIA. Comparative profiling of ICM *versus* control and DCM *versus* control showed 1081 and 2440 differentially expressed genes, respectively (>1.29-fold; *P*<0.05). GeneMANIA revealed differentially regulated functional networks specific to ICM and DCM. In comparison with CNT, differential expression was seen in 9 and 12 nucleocytoplasmic transport-related genes in ICM and DCM groups, respectively. *DDX3X*, *KPNA2*, and *PTK2B* were related to ICM, while *SMURF2*, *NUP153*, *IPO5*, *RANBP3*, *NOXA1*, and *RHOJ* were involved in DCM pathogenesis. Furthermore, the two pathologies shared 6 altered genes: *XPO1*, *ARL4, NFKB2*, *FHL3*, *RANBP2*, and *RHOU* showing an identical trend in expression in both ICM and DCM. Notably, the core of the derived functional networks composed of nucleocytoplasmic transport-related genes (*XPO1*, *RANBP2*, *NUP153*, *IPO5*, *KPNA2*, and *RANBP3*) branched into several pathways with downregulated genes. Moreover, we identified genes whose expression levels correlated with left ventricular mass index and left ventricular function parameters in HF patients. Collectively, our study provides a clear distinction between the two pathologies at the transcriptome level and opens up new possibilities to search for appropriate therapeutic targets for ICM and DCM.

## Introduction

Heart failure (HF) is one of the most common health disorders, which incurs very costly treatments in developed countries. Nevertheless, HF remains a major health problem, with high prevalence and poor prognosis [1]. This condition arises because of an abnormality in cardiac structure, function, rhythm, or conduction [2]. In recent years, the elucidation of transcriptome complexity of an organism and understanding the underlying functions of various differentially expressed genes have become a major focus for post-genome research. Beside the classical microarray approach for profiling transcripts, the recent development of RNA-Sequencing (RNA-seq) has revolutionized the studies of whole transcriptomes, providing potentially unlimited measure of all transcripts and splicing variants that are expressed in a cell type [3], [4]. However, the analysis and interpretation of the huge amount of data generated by RNA-Seq poses a practical challenge and demands accurate and easily automated bioinformatic tools for processing data sets [5].

Thus far, only a few studies have revealed unique cardiac transcriptomic signatures associated with HF using deep RNA-Seq [6], [7]. Others have employed RNA-Seq in conjunction with other techniques to obtain a more comprehensive molecular characterization of HF [8], [9]. Despite the emerging data on RNA-Seq, a clear differentiation between the two major causes of HF, ICM and DCM, based on their transcriptome profiles has not been established yet by this approach.

With the availability of immense amount of genome-wide expression profiling data sets, data-mining algorithms for deriving *in silico* gene functional interpretations relevant to a particular disease state or experimental condition have become an integral part of almost all data analyses. In the present study, we employed GeneMANIA (Gene Multiple Association Network Integration Algorithm), which is a rapid and accurate heuristic algorithm that builds a composite functional association network by integrating multiple functional association networks and predicts gene function in real-time. It identifies other genes that are related to a set of input genes, using a very large set of functional interaction data [10], [11], and thus aids in generating hypotheses about gene function, analyzing gene lists and prioritizing genes for functional assays.

Our group has lately focused on nucleocytoplasmic transport studies, describing alterations in the nucleocytoplasmic trafficking machinery [12], the levels and distribution of components of the nuclear pore complex [13], and changes in nucleocytoplasmic-related gene expression in an earlier microarray-based study [14].

Therefore, in view of the above and a paucity of data on transcriptome profiling by RNA-Seq, the objective of our study was to simultaneously profile the transcriptomes of both ICM and DCM by using RNA-Seq, investigate the nucleocytoplasmic transport-linked functional network underlying the two pathologies, and further analyze the correlation between the mRNA levels of these genes and left ventricular dysfunction.

## Methods

### Tissue samples

Left ventricular tissue samples were obtained from 31 subjects: 13 patients with ICM, and 10 patients with DCM undergoing cardiac transplantation. 8 non-diseased donor hearts were used as CNT samples. Clinical history, ECG, Doppler echocardiography, hemodynamic studies, and coronary angiography data of the patients were available. ICM was diagnosed on the basis of the clinical history, Doppler echocardiography, and coronary angiography data. Non-ischemic DCM was diagnosed when patients had left ventricular systolic dysfunction (EF<40%) with a dilated non-hypertrophic left ventricle (LVEDD>55 mm) on echocardiography. Moreover, patients did not show the existence of either primary valvular disease or familiar DCM. The clinical characteristics and comorbidities of the patients are shown in Table 1. All patients were functionally classified according to the NYHA criteria and were receiving medical treatment following the guidelines of the European Society of Cardiology [15].

**Table 1:** Clinical characteristics of patients according to HF etiology.

	ICM	DCM
	(n = 13)	(n = 10)
**Age (years)**	54±7	54±9
**Gender male (%)**	100	90
**NYHA class**	3.5±0.4	3.3±0.3
**BMI (kg/m^2^)**	26±4	27±7
**Hemoglobin (mg/mL)**	14±3	13±3
**Hematocrit (%)**	41±6	39±8
**Total cholesterol (mg/dL)**	162±41	139±30
**Prior hypertension (%)**	30	11
**Prior smoking (%)**	84	22
**Prior diabetes mellitus (%)**	38	18
**EF (%)**	24±4*	18±6
**FS (%)**	13±2*	10±3
**LVESD (mm)**	55±7*	68±12
**LVEDD (mm)**	64±7*	76±11
**LV mass (g)**	262±68*	434±111
**LVMI (g/cm^2^)**	139±36*	239±85

Data are showed as the mean value ± SD. ICM, ischemic cardiomyopathy; DCM, dilated cardiomyopathy; NYHA, New York Heart Association; BMI, body mass index; EF, ejection fraction; FS, fractional shortening; LVESD, left ventricular end-systolic diameter; LVEDD, left ventricular end-diastolic diameter; LV mass, left ventricular mass; LVMI, left ventricular mass index.

*P<0.05 significantly different between ICM and DCM patients.

Eight non-diseased donor hearts were used as CNT samples, which could not be transplanted because of blood type or size incompatibility. The cause of death in these individuals was cerebrovascular or motor vehicle accident. All donors had normal left ventricular function and no history of myocardial disease or active infection at the time of transplantation.

All heart samples were obtained with informed written consent of patients or their families. The project was approved by the Ethics Committee (Biomedical Investigation Ethics Committee of La Fe University Hospital of Valencia, Spain), and conducted in accordance with the guidelines of the Declaration of Helsinki [16].

Left ventricle samples were collected from near the apex and maintained in 0.9% NaCl at 4°C for a maximum of 6 h following coronary circulation loss, and then stored at −80°C until RNA extraction.

### RNA extraction

Heart tissue samples were homogenized using TRIzol agent in a TissueLyser LT apparatus (Qiagen, UK). RNA was extracted using the PureLink Kit according to the manufacturer's recommendations (Ambion, Life Technologies, CA, USA). RNA was quantified using a NanoDrop1000 spectrophotometer (Thermo Fisher Scientific, UK), and the purity and integrity of RNA samples were measured using a microfluidics-based platform 2100 Bioanalyzer with the RNA 6000 Nano LabChip Kit (Agilent Technologies, Spain). All RNA samples displayed a 260/280 absorbance ratio ≥2.0, and RNA integrity numbers were ≥9.

### RNA-Seq analysis

Poly(A) RNA samples were isolated from 25 µg of total RNA using the MicroPoly(A) Purist kit (Ambion, USA). SOLiD 5500 XL platform was used for sequencing whole transcriptome libraries generated from total Poly(A) RNA samples, following the manufacturer's instructions (Life Technologies, CA, USA). No RNA-spike in CNTs was used. Amplified cDNA quality was analyzed using the Bioanalyzer 2100 DNA 1000 kit (Agilent Technologies, Spain), and quantified using the Qubit 2.0 Fluorometer (Invitrogen, UK). The whole transcriptome libraries were used for making SOLiD-templated beads following the SOLiD System Templated Bead Preparation guidelines. This protocol comprised a clonal amplification step following an enrichment and chemical modification process. Bead quality was estimated based on workflow analysis parameters. The samples were sequenced using the 50625 paired-end protocol, generating 75 nt + 35 nt (paired-end) + 5 nt (barcode) sequences. Quality data was measured using SOLiD Experimental Tracking Software parameters.

### RNA-Seq data computational analysis

The initial whole transcriptome paired-end reads obtained from sequencing were mapped against the latest version of the human genome (version GRchr37/hg19) using the Life Technologies mapping algorithm (version 1.3, http://www.lifetechnologies.com/). The aligned records were reported in BAM/SAM format [17]. Insufficient quality reads (Phred score <10) were eliminated using the Picard Tools software (version 1.83, http://picard.sourceforge.net/). Gene predictions were estimated using the Cufflinks method [18], and the expression levels were calculated using the HT Seq software (version 0.5.4p3, http://www-huber.embl.de/users/anders/HTSeq/). This method employs unique reads for the estimation of gene expression and eliminates the multimapped reads.

### Statistical analysis

Differential expression analysis between conditions was assessed using edgeR method (version 3.2.4) [19]. This method relies on different normalization process-based in depth global samples, CG composition, and length of genes (http://www.bioconductor.org/), and is based on a Poisson model to estimate the variance of the RNA-Seq data for differential expression. Finally, we selected differentially expressed genes with a *P* value <0.05 and a fold change of at least 1.29. The data discussed in this publication have been deposited in NCBI's Gene Expression Omnibus (GEO) [20], and are accessible through GEO Series accession number GSE55296 (http://www.ncbi.nlm.nih.gov/geo/query/acc.cgi?acc=GSE55296).

### GeneMANIA

GeneMANIA (version 3.2.1, http://www.genemania.org/) analysis of the differentially expressed genes related to nucleocytoplasmic transport was performed between CNT and ICM/DCM (>1.29-fold; *P*<0.05). It finds genes that are related to a set of input genes, using a very large set of functional interaction data [10], [11]. We performed gene network analysis to identify gene–gene interactions, study the topology of this gene correlation between the two comparisons, and predict additional genes that may be involved in ICM/DCM if they are shown to interact with a large number of genes in the query set. In the present study, the association data of GeneMANIA algorithm was selected from the pathway and the protein-protein interaction databases. The interactions based on the known protein domains, co-localization, and co-expression profiles were eliminated from the analysis, as this information could increase the false-positive ratio in the resultant functional network.

## Results

### Clinical characteristics of patients

Using RNA-Seq, we analyzed 31 human hearts, out of which 23 were explanted human hearts from patients diagnosed with HF undergoing cardiac transplantation and 8 were non-diseased donor hearts as CNT samples. All analyzed patients were men, except for one woman in the DCM group. The mean age of the patients was 54±8 years. The patients had a New York Heart Association functional classification of III–IV and had previously been diagnosed with significant comorbidities, including hypertension and hypercholesterolemia. The CNT group had a mean age of 49±17 years and 80% of them were men.

Table 1 shows the mean ± SD of the clinical characteristics of the patients according to the etiology of HF. The ICM group had significantly higher values for ejection fraction (EF) and fractional shortening (*P*<0.05). Notably, the parameters left ventricle end-systolic diameter (LVESD), left ventricle end-diastolic diameter (LVEDD), left ventricle mass, and left ventricular mass index (LVMI) were significantly lower in the ICM group when compared to those in the DCM group (*P*<0.05).

### Gene expression analysis by RNA-Seq

To investigate the changes accompanying human HF, we performed a large-scale expression screen in 31 heart samples (ICM, n = 13; DCM, n = 10; and CNT, n = 8) by using RNA-Seq technology. Significant analysis of the RNA-Seq results revealed a total of 1081 genes that were differentially expressed in ICM patients *vs.* CNT (>1.29-fold; *P*<0.05), of which 823 were upregulated and 258 downregulated. Additionally, 2440 genes were differentially expressed in DCM patients *vs.* CNT (>1.29-fold; *P*<0.05), being 956 genes upregulated and 1484 donwregulated (Table S1).

**Figure 1:**
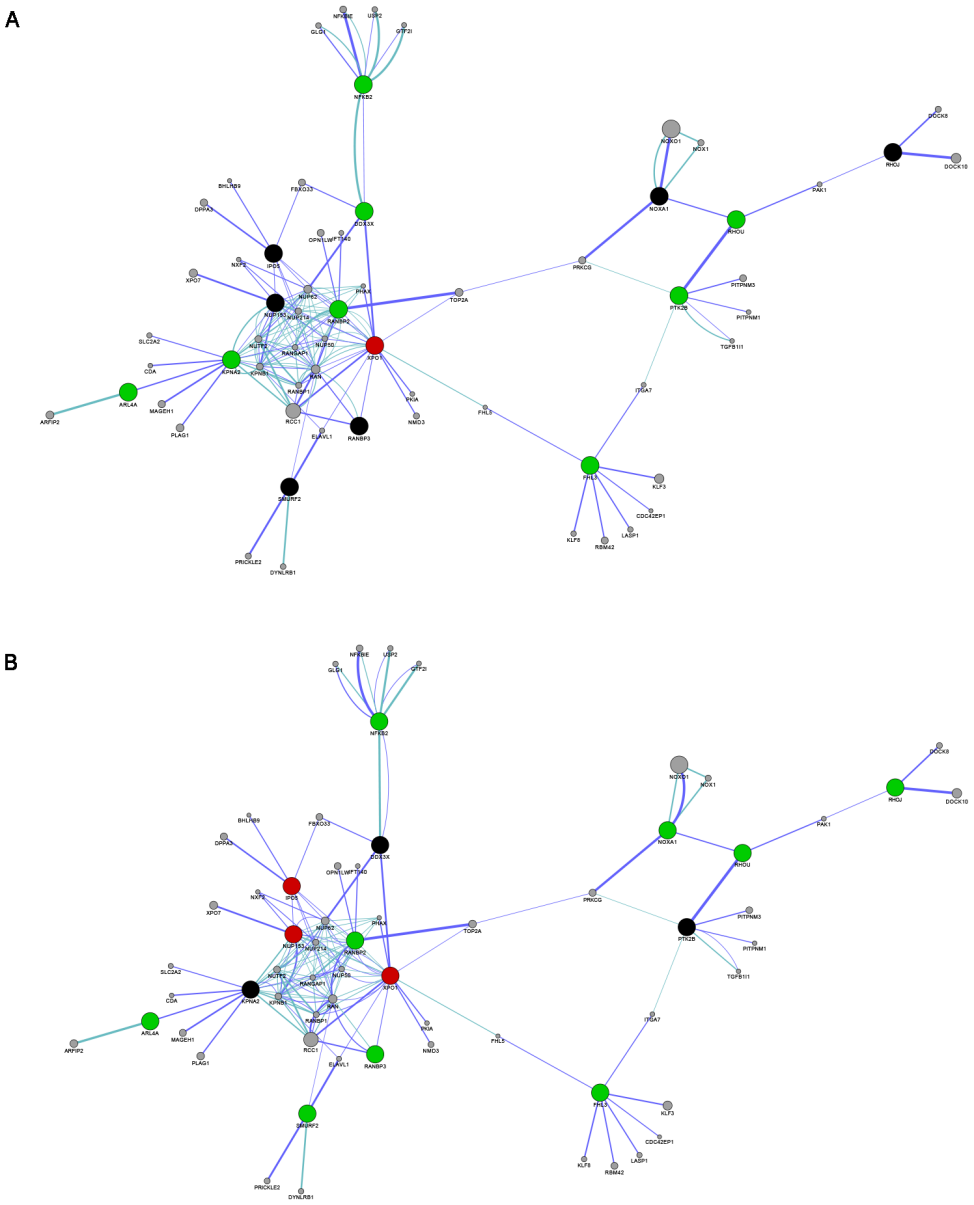
Functional network for ICM (A) and DCM (B). Analysis was based on protein-protein interaction (dark blue lines) and pathway (light blue lines) databases with the nucleocytoplasmic transport-related genes (>1.29-fold; *P*<0.05). GeneMANIA retrieved known and predicted interactions between these genes and added extra genes (small grey circles) that are strongly connected to query genes (large circles). Red indicates upregulation, green indicates downregulation and black indicates no differences in the trend expression between the two comparisons (ICM *vs*. CNT and DCM *vs*. CNT).

Since our study focused on the nucleocytoplasmic transport process, we used the GeneMANIA algorithm to analyze the transcriptome alterations related to this functional category among the differentially expressed genes in both pathologies in comparison with the CNT. Thus, a seed gene list related to this functional category was obtained to achieve a highly specific correlation (protein interaction, pathways) among the transport genes (Table 2). Figure 1 shows the functional network of genes obtained in this study, besides the nucleocytoplasmic transport–related genes.

**Table 2:** Gene list of nucleocytoplasmic transport-related genes (>1.29-fold; *P*<0.05) used as query genes in GeneMANIA.

		Fold change	p-value	Description
**ICM**	***XPO1***	1.64	0.000035	Exportin 1
	***KPNA2***	−1.35	0.028475	Karyopherin alpha 2 (RAG cohort 1, Importin alpha 1)
	***PTK2B***	−1.47	0.026496	Protein tyrosine kinase 2 beta
	***DDX3X***	−1.50	0.002262	DEAD (Asp-Glu-Ala-Asp) box helicase 3, X-linked
	***FHL3***	−1.51	0.020446	Four and a half LIM domains 3
	***RANBP2***	−1.53	0.000435	RAN binding protein 2
	***NFKB2***	−1.61	0.004864	Nuclear factor of kappa light polypeptide gene enhancer in B-cells 2
	***ARL4A***	−1.61	0.006728	ADP-ribosylation factor-like 4A
	***RHOU***	−1.77	0.015125	Ras homolog family member U
**DCM**	***XPO1***	1.69	0.000007	Exportin 1
	***IPO5***	1.32	0.010064	Importin 5
	***NUP153***	1.30	0.040878	Nucleoporin 153 kDa
	***RANBP3***	−1.29	0.013812	RAN binding protein 3
	***FHL3***	−1.36	0.019077	Four and a half LIM domains 3
	***SMURF2***	−1.40	0.010070	SMAD specific E3 ubiquitin protein ligase 2
	***RANBP2***	−1.42	0.001289	RAN binding protein 2
	***RHOJ***	−1.56	0.000756	Ras homolog family member J
	***NOXA1***	−1.64	0.011258	NADPH oxidase activator 1
	***ARL4A***	−1.81	0.004950	ADP-ribosylation factor-like 4A
	***RHOU***	−1.83	0.032471	Ras homolog family member U
	***NFKB2***	−1.92	0.000004	Nuclear factor of kappa light polypeptide gene enhancer in B-cells 2

In comparison with the CNT group, differential expression was seen in 9 genes related to ICM and 12 genes related to DCM. Furthermore, the two pathologies shared 6 altered genes: *XPO1* (Exportin 1), *ARL4* (ADP-ribosylation factor-like 4A), *NFKB2* (Nuclear factor of kappa light polypeptide gene enhancer in B-cells 2), *FHL3* (Four and a half LIM domains 3), *RANBP2* (RAN binding protein 2), and *RHOU* (Ras homolog family member U) that showed an identical trend in expression in both ICM and DCM. Collectively, our findings revealed that the genes *DDX3X* (DEAD (Asp-Glu-Ala-Asp) box helicase 3, X-linked), *KPNA2* (Karyopherin alpha 2 (RAG cohort 1, Importin alpha 1), and *PTK2B* (Protein tyrosine kinase 2 beta) were exclusively related to ICM, while *SMURF2* (SMAD specific E3 ubiquitin protein ligase 2), *NUP153* (Nucleoporin 153 kDa), *IPO5* (Importin 5), *RANBP3* (RAN binding protein 3), *NOXA1* (NADPH oxidase activator 1), and *RHOJ* (Ras homolog family member J) were involved in DCM.

In addition, the core of the derived functional network was composed of the nucleocytoplasmic transport-related genes, *XPO1*, *RANBP2*, *NUP153*, *IPO5*, *KPNA2*, and *RANBP3*, which are involved in both pathologies (Figure 1). Interestingly, it was found that all branch-point genes arising from the core were downregulated.

### Relationship between gene expression and echocardiographic parameters

We determined if there was any relationship between the expression of the studied genes and the clinical characteristics shown in Table 1. While LVMI was found to be directly associated with the expression of *DDX3X* (r = 0.727, *P* = 0.017) and inversely related with the expression of *NFKB2* (r = −0.643, *P* = 0.045) and *FHL3* (r = −0.765, *P* = 0.01) in the ICM group, a significant positive relationship was observed with the expression of *ARL4* (r = 0.776, *P* = 0.040) and *NFKB2* (r = 0.769, *P* = 0.044) in the DCM group. Moreover, EF showed an inverse significant correlation with *XPO1* expression (r = −0.643, P = 0.045) in the ICM group (Table 3).

**Table 3:** Correlations between gene expression and echocardiographic parameters (*P*<0.05).

	ICM				DCM	
	*XPO1*	*DDX3X*	*NFKB2*	*FHL3*	*ARL4*	*NFKB2*
**LVMI**		r = 0.762	r = −0.643	r = −0.710	r = 0.776	r = 0.769
**FE**	r = −0.643					

## Discussion

In the present study, RNA-Seq-based global transcriptome analysis was performed to compare the transcriptome profiles of HF patients (with ICM or DCM) undergoing heart transplantation with healthy controls. Further, we employed the GeneMANIA algorithm to analyze the transcriptome alterations related to nucleocytoplasmic transport in both pathologies in comparison with the CNT.

RNA-Seq identified 1081 genes in ICM and 2440 genes in DCM to be differentially expressed in comparison with the CNT (>1.29-fold; *P*<0.05). Subsequently, the GeneMANIA algorithm was used to predict the functions of the differentially expressed genes in both pathologies based on the transcriptome alterations related to the nucleocytoplasmic transport, in comparison with the CNT. Further, we deduced a highly specific functional network composed of a total of 9 differentially expressed genes in ICM, which included 1 upregulated and 8 downregulated genes, and 12 differentially expressed genes in DCM with 3 upregulated and 9 downregulated genes.

Interestingly, the core of the functional network was composed of nucleocytoplasmic transport genes such as the exportin *XPO1*, the importins *KPNA2* and *IPO5*, the nucleoporin *NUP153*, and the Ran-binding proteins *RANBP2* and *RANBP3*. Notably, the branch-point genes arising from the core, all of which are related to the nucleocytoplasmic transport were found to be downregulated.

Taken together, these data suggest that in ICM and DCM, the nucleocytoplasmic transport is altered, thus initiating the inhibition of different pathways in cardiomyocytes. These inhibited pathways are composed of genes involved in a variety of cellular processes that code for a transcription factor (*NFKB2*), an RNA helicase (*DDX3X*), Arf-like GTPase (ARL4), E3 ubiquitin ligase (*SMURF2*), a transcriptional coactivator and cytoskeleton regulator (*FHL3*), a cytoplasmic tyrosine kinase (*PTK2B*), a protein that activates NADPH oxidases (*NOXA1*), a member of the Rho family of GTPases (*RHOU*), and small GTP-binding proteins in the Rho family (*RHOJ*). Furthermore, the 6 genes from the network (*XPO1*, *ARL4*, *NFKB2*, *FHL3*, *RANBP2*, and *RHOU*) showed the same trend in expression compared to the CNT group in both pathologies. Therefore, *DDX3X*, *KPNA2* and *PTK2B* are involved in the pathogenesis of ICM, while *SMURF2*, *NUP153*, *IPO5*, *RANBP3*, *NOXA1* and *RHOJ* characterize DCM.

In the present study, *DDX3X* expression was found to be downregulated in ICM. DDX3X interacts with the exportin XPO1, and it is localized in the cytoplasmic side of the nuclear pore complex. DDX3X belongs to the DEAD-box proteins, a large family of ATP-dependent RNA helicases. A study by Yedavally *et al.* suggested that DDX3X could be the human RNA helicase, which functions in the XPO1 RNA export pathway analogously to the postulated role for Dbp5 in yeast mRNA export [21]. Moreover, DDX3X has been shown to upregulate the levels of the transcription factor Snail [22], whose nuclear export is mediated by an XPO1-dependent mechanism [23], and it further enhances the translation of HIV-1 gRNA in a nuclear export-dependent manner through XPO1 [24]. Thus, the function of DDX3X and XPO1 is closely linked. Therefore, collectively these data suggest that DDX3X may not be the preponderant RNA helicase in the mechanism underlying ICM pathogenesis, although the nucleocytoplasmic transport is increased in ICM.

A similar behavior was noted in *RANBP3* presumed to be involved in the DCM. *RANBP3* encodes a RAN-binding protein that shuttles between the nucleus and the cytoplasm by an XPO1-dependent mechanism. It has been shown that RANBP3 binds directly to XPO1 acting as a cofactor, thereby enhancing the affinity between Ran:GTP and cargo [25]. In addition, RANBP3 acts as a scaffold protein to promote the efficient assembly of XPO1-dependent export complexes [26]. However, in our study, the expression of *RANBP3* was found to be downregulated, indicating that it is not the pre-eminent XPO1 cofactor in DCM.

Strikingly, the only genes whose expression was higher in the DCM functional network were the importin, *IPO5*, and the nucleoporin, *NUP153*. A previous study by our group showed that the protein levels of IPO5 and NUP153 increase in patients with HF [12], [13], and thus, these proteins could be predominantly controlling the nucleocytoplasmic transport in DCM, in conjunction with XPO1. To further strengthen this fact, it has been reported that both IPO5 and NUP153 control the nucleocytoplasmic transport of the RNA binding protein CPEB3 [27]. Therefore, it is evident that exists a strong interaction amongst XPO1, IPO5, and NUP153 whilst controlling the nucleocytoplasmic transport in DCM.

Furthermore, in ICM functional network *KPNA2* and *PTK2B* were found to be significantly downregulated. *KPNA2* encodes an importin involved in nuclear transport [28], highly abundant and capable of binding a variety of import signals [29]. It has been described that in addition to the selectivity for different cargos, the differential expression of *KPNA2* during development and differentiation presents an important regulatory mechanism [30], and that the transport of factors to the nucleus through KPNA2 allows its cellular function to be fine-tuned, such as, the interaction of KPNA2 with the kinase ASK1 in response to stress [31]. Therefore, since KPNA2 is involved in the transport of molecules under a wide variety of cell conditions, it could be relevant in ICM taken into account the stress conditions that exists in this cardiomyopathy. Our group has previously described that the gene expression of *XPO1* increases in ICM [14]. Consequently, both the exportin XPO1 and the importin KPNA2 seem to play a major role in ICM.

It is well known that *PTK2B* gene encodes the cytoplasmic protein tyrosine kinase, Pyk2 that is involved in calcium-induced regulation of ion channels including K^+^ and Ca^2+^ channels and activation of the mitogen-activated protein kinase signaling pathway [32]–[34]. Pyk2 expression was first reported in human hearts by Lang *et al*, who demonstrated Pyk2 to be significantly activated in non-ischemic, but not in ICM, and that its expression remained constant across disease states including end stage HF [35]. Consistent with the above findings, our results showed a significant downregulation of *PTK2B* only in ICM. Furthermore, a study by Hart *et al* suggested the role of Pyk2 in promoting the deterioration of the left ventricular remodeling post- myocardial infarction wherein, the adenovirus-mediated expression of a dominant negative inhibitor of Pyk2 signaling after myocardial infarction (MI) in rats resulted in improved survival, increased LV function, and altered expression of myosin heavy chain isozymes, indicating an attenuation of LV remodeling post-MI [36].

Notably, the genes whose expressions were also found to be downregulated in the DCM functional network were *RHOJ*, *NOXA1*, and *SMURF2*. *RHOJ* encodes one of the many small GTP-binding proteins in the Rho family, and was shown to be associated with focal adhesions in endothelial cells [37], [38]. A novel coexpression and integrated pathway network analysis revealed that *RHOJ* plays a central role in the pathophysiology of murine progressive cardiomyopathy [39]. Thus, *RHOJ* gene is likely to play a central role in the pathophysiology of DCM.

*NOXA1* is a critical component of the vascular smooth muscle cells NADPH oxidase [40]. NoxA1 expression is correlated with the progression of atherosclerotic lesions and modulates the NADPH oxidase activity under pathophysiological conditions [41]. Although Nox enzymes participate in a broad array of cellular functions, the role of Nox1 enzymes in cardiovascular disease has been studied mainly in hypertension [42]. Therefore, these findings indicate a plausible role of *NOXA1* in the regulation of DCM pathogenesis.

*SMURF2* encodes an E3 specific ubiquitin protein ligase that negatively regulates the TGFβ (transforming growth factor β) signaling pathway, which determines embryogenesis and tissue homeostasis, through a negative feedback mechanism, and controls the strength and duration of the signal transduction [43], [44]. Hence, *SMURF2* might act as a master regulator of this pathway in DCM orchestrating its regulation by ubiquitination.

Left ventricular remodeling is the process by which ventricular size, shape, and function are regulated by mechanical, neurohormonal, and genetic factors [45]–[46]. Remodeling may be deleterious and is generally accepted as a determinant of the clinical course of HF [47]. Patients with major remodeling demonstrate progressive worsening of cardiac function, influencing the course of the heart disease. We determined whether there were any correlations between the expression of the genes comprised in the networks and the echocardiographic parameters included in Table 1. We found a relationship between LVMI and the genes *DDX3X*, *NFKB2*, and *FHL3* in the ICM group, indicating that higher levels of *DDX3X* and lower levels of *NFKB2* and *FHL3* are linked with cardiac function impairment in ICM. On the other hand, we also found a direct correlation between LVMI and the genes *ARL4* and *NFKB2* in the DCM group. It is interesting to point out that *NFKB2* gene presents an opposite correlation in ICM and DCM. This could be attributed to the fact that *NFKB2* encodes a subunit of the transcription factor complex nuclear factor-kappa-B (NFκB), which could function as both a transcriptional activator and repressor depending on its dimerization partner [48], [49], and that the transcription factor complex NFκB when activated by a myriad of stimuli exerts its transcriptional effects on upwards of 150 genes [50].

In addition, in the ICM group, EF showed a significant inverse correlation with *XPO1*, highlighting a significant link between the left ventricular function and the expression of this gene, as it was reported in a previous study of our group [14]. However, not all significant correlations obtained between *XPO1* and relevant left ventricular parameters in ICM could be established in this study, maybe due to the small sample size.

In summary, our findings suggest the existence of an expression network signature in HF diseased hearts, offering important insights into the pathogenesis of this condition. This study is based on high-throughput dataset and functional network analysis that pinpoints at genes relevant for HF in both ICM and DCM. Interestingly, the networks are composed of a core of nucleocytoplasmic transport related genes that give rise to several downregulated pathways. Understanding the regulation of the expression of these genes may provide potential targets for therapeutic intervention.

## Supporting Information

Table S1 **Significantly altered genes in ICM and DCM pathologies (>1.29-fold;** ***P*** **<0.05).**
(XLSX)
